# Social mixing and network characteristics of COVID-19 patients before and after widespread interventions: A population-based study

**DOI:** 10.1017/S0950268823001292

**Published:** 2023-08-14

**Authors:** Yuncong He, Leonardo Martinez, Yang Ge, Yan Feng, Yewen Chen, Jianbin Tan, Adrianna Westbrook, Changwei Li, Wei Cheng, Feng Ling, Huimin Cheng, Shushan Wu, Wenxuan Zhong, Andreas Handel, Hui Huang, Jimin Sun, Ye Shen

**Affiliations:** 1 School of Mathematics, Sun Yat-sen University, Guangzhou, China; 2 Department of Epidemiology, School of Public Health, Boston University, Boston, USA; 3 School of Health Professions, University of Southern Mississippi, Hattiesburg, USA; 4 Zhejiang Provincial Center for Disease Control and Prevention, Hangzhou, China; 5 Department of Epidemiology and Biostatistics, College of Public Health, University of Georgia, Athens, USA; 6 Department of Epidemiology, Tulane University School of Public Health and Tropical Medicine, New Orleans, USA; 7 Department of Statistics, University of Georgia, Athens, USA; 8 Center for Applied Statistics and School of Statistics, Renmin University of China, Beijing, China

**Keywords:** COVID-19, network, temporal, widespread intervention, agent-based simulation

## Abstract

SARS-CoV-2 rapidly spreads among humans via social networks, with social mixing and network characteristics potentially facilitating transmission. However, limited data on topological structural features has hindered in-depth studies. Existing research is based on snapshot analyses, preventing temporal investigations of network changes. Comparing network characteristics over time offers additional insights into transmission dynamics. We examined confirmed COVID-19 patients from an eastern Chinese province, analyzing social mixing and network characteristics using transmission network topology before and after widespread interventions. Between the two time periods, the percentage of singleton networks increased from 38.9 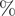 to 62.8 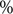 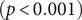 ; the average shortest path length decreased from 1.53 to 1.14 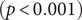 ; the average betweenness reduced from 0.65 to 0.11 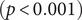 ; the average cluster size dropped from 4.05 to 2.72 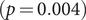 ; and the out-degree had a slight but nonsignificant decline from 0.75 to 0.63 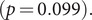 Results show that nonpharmaceutical interventions effectively disrupted transmission networks, preventing further disease spread. Additionally, we found that the networks’ dynamic structure provided more information than solely examining infection curves after applying descriptive and agent-based modeling approaches. In summary, we investigated social mixing and network characteristics of COVID-19 patients during different pandemic stages, revealing transmission network heterogeneities.

## Introduction

SARS-CoV-2 is characterised by a high transmission rate and has rapidly spread globally [1, 2]. Transmission of SARS-CoV-2 is largely dependent on social network structures and clinical characteristics of people with disease [3]. Despite this, transmission networks in the context of SARS-CoV-2 and how they have changed throughout the pandemic have been poorly and insufficiently described [4–7].

With a robust and comprehensive COVID-19 surveillance system in Zhejiang Province, China, we collected individual-level data of all confirmed COVID-19 cases in Zhejiang, China [8, 9]. All cases with directed epidemiological linkage form a transmission network (see *Data Source* for details). Thus, we utilised such data to further our understanding of transmission networks and how they change over time. We aimed to report COVID-19 transmission network characteristics, graphical structures of disease networks, and how networks were impacted by and associated with nonpharmaceutical interventions (NPIs), including social distancing, active case finding, and controlling policy for household members.

Through COVID-19 case investigations, patients’ transmission network and their social characteristics were collected. We first reported on all observed network links and then explored the dynamics of network topological structure (before and after widespread interventions). To illustrate whether network structures are closely related to disease-spreading processes, we then built network models to simulate underlying transmission processes by incorporating social contact strength [10, 11], age distribution [12, 13], and family contribution of symptomatic cases [14, 15]. With these network models, simulations were constructed to study dynamic associations between the development of an epidemic and the transmission network structure under distinct scenarios. Through field investigations, we identified heterogeneities in the dynamic changes in the transmission network structure associated with social characteristics. Then, we conducted a comprehensive exploration of the mechanism through advanced modelling techniques and found that interventions may regulate infection risk based on social structures. We anticipated that our results will provide guidance for future outbreak control.

## Materials and methods

### Data source

We collected individual-level data on 1,349 SARS-CoV-2 infections during the COVID-19 pandemic in Zhejiang Province, China, from 8 January to 23 February 2020 (see *Data source* in the Supplementary materials for more details). The data contain detailed information about each diagnosed case such as their personal information (age, gender, family, occupation location, the severity of infection, imported cases or not, date of symptom onset, and date of laboratory confirmation) and, more critically, their potential exposures, that is epidemiological linkage to other cases identified through contact tracing efforts. Here, we assumed that there was no re-infection (i.e. one cannot be infected by SARS-CoV-2 more than once in a relatively short period) and also no co-infection (because there is only one source case for each infected patient). The infector–infectee transmission pairs constructed the transmission networks. Edges directly connecting individuals (called nodes) formed a cluster. Each node and edge inside had heterogeneity with respect to their social features. The topological characteristics of the networks changed with time. To better understand the patterns and drivers of such heterogeneity, we analysed the network quantitatively.

### Description of network features

To quantitatively describe the networks, we measured the structure of five key characteristic parameters: (a) out-degree (number of secondary cases); (b) shortest path length (number of directed steps along the shortest paths between network nodes); (c) betweenness centrality [16] (how important a node is in terms of connecting other nodes [17]); (d) diameter of clusters (generations of the spreading); and (e) cluster size (see *Methods* in Supplementary Material for more formal, detailed definitions [18–21]).

**Figure 1.**
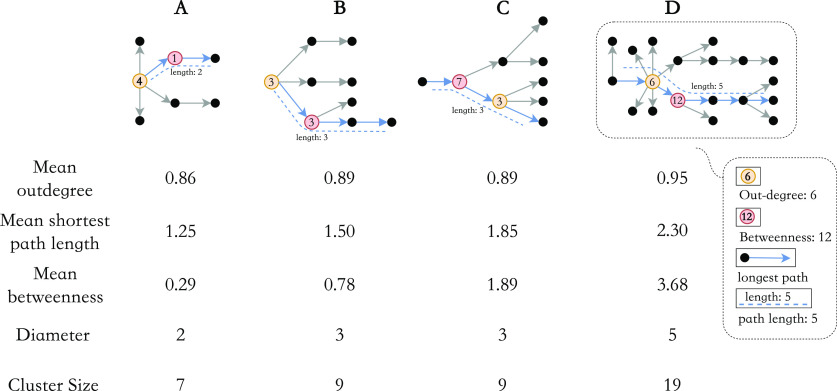
Observed clusters (A and D) and hypothetical examples (B and C) and their respective basic graphical measures. The average shortest path length represents the ability of a network to expand and extend, and the average betweenness measures the average level of contribution for disease spreading. In each cluster, we highlighted nodes and paths contributing greatly to the viral transmission, which is reflected by their high out-degree and high betweenness centrality (i.e. node importance) and longest path length (i.e. path importance). Hence, the clusters decrease in extensibility from cluster D to cluster A due to reduced branching, and the number and influence of central cases in disease transmission also decline.

The average shortest path length between nodes measures how far one node is from another and thus can be used to quantify the connectivity of a network. Typically, for a directed transmission network, the average shortest path length characterises its ability to expand and extend. Networks with a shorter average shortest path length tend to have fewer branches along the transmission chain and are also less likely to introduce a large infection generation (i.e. shorter transmission chains). Betweenness centrality measures the importance of a node in terms of connecting other nodes [17], that is for disease spreading. If nodes with high betweenness centrality were removed from the network, the network would have diminished. In Figure 1, we provided four illustrative examples to demonstrate how these measures capture the presence of additional branches and longer chains in a transmission network and identify the nodes with higher reproductive potential. We could observe clusters A and D from our data, while clusters B and C were conceptual.

We also defined superspreaders as cases with an out-degree of at least three (which is the 95% quantile of the distribution of out-degree; see Figure 4). By considering in-degree (1 or 0 in a transmission network; the source of an individual’s infection) and out-degree information, we could define singletons (isolated nodes), index cases (the source of each cluster), and terminal cases (the terminal node of each cluster). The mathematical definitions and key concepts in a network analysis such as nodes, edges, singletons, clusters, and other network quantities are described in detail in the Supplementary materials.

To perform a comparison of topological characteristics of the transmission network across time, we chose a time point to split the network into two periods. Zhejiang provincial government upgraded its infectious disease alert category to the highest level on 23 January 2020, began an officially comprehensive set of restrictions on 1 February 2020, and started reopening on 10 February. Before the governmental announcement, however, on 20 January evidence of human-to-human transmission was already confirmed and raised public awareness. Consequently, outbreaks in Wuhan and reports from news media had already triggered nationwide voluntary social distancing and personal protection before 23 January. Another key factor was Chinese New Year’s Eve on 24 January. Schools had already been on holiday for a while, and work-related social contacts were at very low levels with the majority of the population taking vacations. Therefore, we believed that before 23 January, the average contact numbers had already dropped significantly and achieved the lowest level shortly after 23 January [22]. Hence, we split the overall network by the declaration date (23 January) to study topological changes in the transmission network (Figure 5).

### Statistical analysis

Arithmetic means and proportions were used for count/ordinal and binary variables, respectively. We used cluster-based bootstrapping [23] to estimate the uncertainty of statistics and conduct hypothesis tests. In particular, we performed 1000 iterations of re-sampling from clusters (i.e. sub-networks that are separated from one another) with replacement to create bootstrapped samples. These samples were then disjointly rejoined to create a pseudo-network sample, and the quantity of interest was calculated for each of these samples. We tested the difference between ordinal variables with Student’s 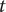 -test and 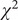 test and binary variables with two proportion 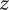 -test [24]. To control the false discovery rate in multiple testing, the Benjamini–Hochberg procedure [25] was used to adjust the 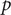 -values. Nonparametric bootstrap percentiles are used to obtain the 95% confidence interval of each quantity. The 95% confidence interval is then estimated by the 2.5 and 97.5 percentiles of the 100 results.

### Agent-based transmission network model

Informed by the observed data, we generated a pseudo-transmission network to comprehensively study the heterogeneous dynamics of the spreading of this COVID-19 outbreak. We assumed that the transmission network is built upon a pre-generated weighted social connection network. Edges, representing social connections between individuals, were weighted based on the types of connections: household, geographical, and random social connections. We fixed the structure of the network, only allowing the weight of edges to vary to represent the changing pattern of social connections. Social contacts between individuals were sampled upon the pre-generated social connection network and infectious contacts occurred through these social contacts. We implemented the susceptible-exposed–infectious-removed (SEIR) model, which subdivides individuals into four transition states: susceptible, exposed, infectious, and removed [26], to characterise the transmission dynamics of COVID-19. Moreover, we categorised patients into 14 age intervals with a 5-year length to construct a time-varying age-specific contact pattern based on the contact matrix between age groups from surveys conducted in Shanghai [11].

**Figure 2.**
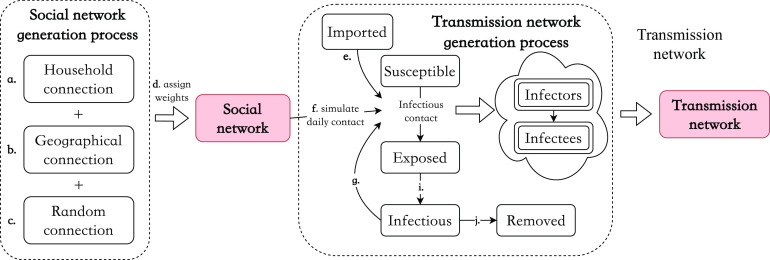
Mechanism of the network generation. The procedures for simulations are marked with a to *k,* respectively.

There are multiple steps for generating the network, which is summarised and labelled in Figure 2, along with key parameters described in Table S1 in the Supplementary Material. In detail, we first generated a social connection network with the following procedures:

- We generated 20,000 nodes (the observed number of close contacts related to 1,349 confirmed cases is approximately 18,000) and distributed them into different families with an average size of 2.95 (see Figure S10 in the Supplementary Material). Each family member was assigned to one of 14 age groups based on the observed distribution of those same 14 age groups in real families of equal size. For individuals residing alone, we randomly assigned their age distribution using the census age distribution in Zhejiang (see Figure S11 in the Supplementary Material, [27]).
- Next, to assign geographic locations to each individual, we randomly sampled a coordinate from a uniform distribution within a square region. Each node in the network could interact with several other nodes, and these interactions were dependent on their respective locations. To be more specific, let 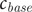 be the age-specific contact matrix in Shanghai before the outbreak and the 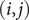 element represented the contact number of the 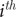 age group with the 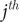 age group. The sum of the 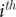 row of the contact matrix was noted as 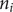 , which represented the average number of contacts per day of a person in the 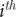 age group. Here, 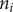 should be less than the number of connections (i.e. the number of acquaintances that one can have), and thus, the 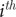 node was assumed to have a connection with 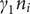 acquaintances. We also normalised the rows of 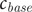 to get the probability of contact of the 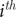 age group with the 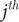 age group, denoted as 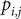 . Accordingly, for an individual in the 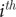 age group, we randomly connected him/her to 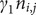 other nodes among the 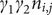 closest nodes of the 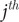 age group, where 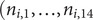 ) were sampled from the multinomial distribution with a total number 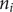 and incident rate 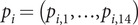 . Here, 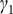 and 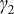 represented the inflation factor to reflect the randomness of the connection. To facilitate contacts from a larger number of acquaintances and allow for relatively long-distance interactions, we set the parameters 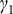 and 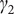 to be equal to 2. Results of a sensitivity analysis indicated that different combinations of parameters (the values of 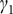 and 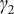 ranging from 1.5 to 3) have little impact on the general structure of the simulated transmission networks, but a small effect on the total number of infections and the proportion of household transmission. This is because the choice of parameters affects the likelihood of long-distance transmissions and infections outside families.
- To reflect the small-world property of the social network [17], we allowed each node to connect with an average of 1 other node at random [4].
- We assigned a weight measure to characterise the importance of different connections [4, 6]. Interventions may impact the transmission network by reducing some contact types (e.g. co-workers under the order of working from home) but risking other types (e.g. household members). We modelled with varied weights of network edges. Typically, weights of size 3.716, 0.5, and 0.5 would be taken for household connection, geographical connection, and random connection, respectively. We raised the weight of the household to 5.041 during the period with the highest-level alert, which ensures that the fraction of contacts at home is 50% during the early outbreak and 79% during that period, based on the contact survey in Shanghai [11]. Combining all the undirected connections above with their weights, we could get a weighted social connection network where each individual connects to their acquaintances, that is the probability of an occurrence of contact is then calculated using a softmax transformation:

(1)

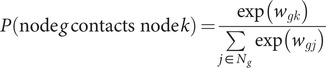

 where 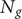 denotes the neighbours of a random node 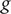 and 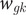 represents the weight between two random nodes 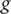 and 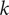 .

Based on the pre-specified weighted social network, we simulated the dynamic transmission network with the following procedures (Figure 2):

1. First, we randomly sampled and inserted 445 nodes among the social network (which is the reported number of imported cases in the epidemic data of Zhejiang) as the index cases into the transmission network, following the same rate of introduction as the observed imported cases (as shown in the Supplementary Material, Figure S8(b)). As soon as an index case is put into the transmission network, it transfers to the infectious state.
2. To reflect the contact patterns that vary by time and age, we considered that the daily contact matrix would initially decrease following the start of the outbreak and gradually return to its previous level once the outbreak was brought under control. The previous and outbreak daily contact matrices were based on the evidence of another study conducted in Shanghai, China [11]. The interventions were applied progressively such that the decline occurred from 10 January, when people began to return home for the Spring Festival, to 23 January, when the provincial government declared a highest-level response. The suppression of social contacts lasted for around one month, until 10 February (the day of the reopening in Zhejiang Province). This was subsequently followed by an increase of up to 50% of the baseline after a month. The details of the construction of the daily contact matrix 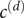 are given in the Supplementary material.
3. In day 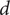 , for a node during the infectious state, we let it contact its neighbours by the weighted social network generated above and following the age-specific contact pattern given by the contact matrix 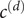 . In detail, we randomly sampled the potential infectees with replacement from the neighbours of an infector in 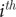 age group on day 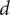 , using a multinomial mass with incident rate calculated from the normalised weight on the equation (1). After that, we randomly added (from its neighbours) or pruned these age-indexed contacts according to the real-time contact vector of 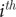 age group with different age groups, which was randomly generated from the multinomial mass with the summation of 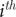 row of 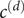 as the total number of contacts and the normalised 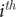 row of 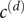 as the incident rate. In particular, if the infector did not connect to any person in some age groups among the pre-specified social network, we repeated the above step until the corresponding real-time contact numbers also meet zero.
4. Infection risk may associate with age [12, 13]. Thus, we assumed an age-specific susceptibility 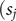 ) (see in the Supplementary Material, Figure S1) according to a previous study [12] and a time-dependent rate of transmissibility (T) from symptom [8] (i.e. transmissibility changes in infectious period and peaks at symptom onset; see Table S3 in the Supplementary Material). The probability of transmission occurring 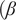 ) for a contact is the product of the common transmissibility and susceptibility based on the age of the potential infectees. If a susceptible node makes contact 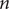 times with an infectious individual on the same day, then the incident probability will be 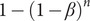 . After being infected, individuals will be assumed to move to the exposed stage.
5. Every infected node during the exposed stage had an incubation period (the duration from being exposed to symptom onset), which is sampled from a log-normal distribution with a log mean of 4.20 and a log standard deviation of 1.94 [28, 29]. We also considered pre-symptomatic infectiousness; that is, infected people can spread the virus to others before the onset of symptoms [29, 30]. We assumed that the duration from exposure to becoming infectious also follows a log-normal pattern and is a random proportion of the incubation period reciprocally following a log-normal distribution with a log mean of 0.04 and a log standard deviation of 0.59. These choices of parameters were chosen to ensure that the duration of the pre-symptomatic infectious period has a mean of 1 [30, 31]. Once an individual becomes infectious, they will transfer to an infectious state. Infected patients commonly produce post-symptomatic viral shedding [29, 32], so a post-symptomatic infectious period is also considered. It is the duration of infectiousness after symptom onset, which is sampled from a gamma distribution with parameters 5 and 1.4 [33]. The overall infectious period is thus 8 days.
6. As long as an individual develops symptoms or is imported on day 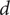 , we assigned it a removal period (duration from symptom onset to being quarantined, reflecting the speed of case finding) according to our observed removal period, which was progressively decreased from above 19 days to below 1 day (Supplementary Material, Figure S8(a)). A node during the infectious state is assumed to transfer to the removed state after the end of the removed period. If the removal period is larger than the corresponding post-symptomatic infectious period, the infected individuals lose infectiousness before being removed or vice versa.
7. For each day 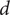 , we recorded the transmission pairs between infectors and infectees. Under the transmission information up to day 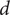 , we were then able to obtain a dynamic transmission network on day 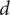 . Eventually, if there are no longer any infectious nodes, the pandemic will stop. For computation considerations, we only simulated 100 days and presented the results in Figures 7 and 8.

### Simulation under population densities, the intensity of social contacts, and levels of case finding

Interventions alter both the pattern of social contact and disease spreading and thereby affect the structure of resulted transmission networks. Therefore, to comprehensively explore the relationship between viral spreading and the dynamics of the resulting transmission network, it is necessary to consider interventions under various settings and intensities. Here, we considered seven scenarios of simulations with various baseline social contact patterns, social distancing intensity, and household contact tracing policy. Based on our network generation setting, we further considered the case with 50% baseline social contact frequency for simulating the outbreak in more sparsely populated areas (denoted as C(L), meaning Contact(Low), as opposite to C(H), meaning Contact(High), which represents the original case). Regarding the social distancing policy, we considered three typical types of intensity: strict lockdown (L(S), meaning Lockdown(Strict)), mild lockdown (L(M)), and no lockdown (L(N)). Compared to baseline contact, they, respectively, represent a contact frequency that drops to 12%, the ratio of contact frequency during the outbreak period (2.3 per day) to the one at the normal time (18.8 per day) in Shanghai, to 50% declination, and to nothing. Compared to a strict active case finding in Zhejiang (R(S)), we considered a relatively mild one (R(M)) with a removal period of at least three days. We also considered a household contact tracing policy (HQ) as an improvement on case finding. When one is confirmed, family members will be tested and quarantined for two weeks at the same time. If a family member is already in an exposed or infectious state, their status will be confirmed, and they will transfer to the removed state. Combining all the considerations above, we set seven scenarios and their settings of parameters are summarised in Table 1. Typically, in a real-data-based scenario (i.e. scenario 1), the Zhejiang provincial government conducted a top-level social distancing policy including quarantining household members of infected individuals and a strictly active case finding.

**Table 1.** Parameters’ setting for seven scenarios with various baseline contact frequency, the intensity of social distancing, and the intensity of active case finding

No.^a^	Baseline social contact^b^	Social distancing	Active case finding	Notes^c^
1	High	Strong and HQ	Strong	Baseline contact high (C(H)) + strict lockdown (L(S)) + strong removal (R(S)) + household quarantine (HQ)
2	High	Mild	Mild	Baseline contact high (C(H)) + mild lockdown (L(M)) + mild removal (R(M))
3	High	Mild	Mild and HQ	Baseline contact high (C(H)) + mild lockdown (L(M)) + mild removal (R(M)) + household quarantine (HQ)
4	Low	NO	Mild	Baseline contact low (C(L)) + no lockdown (L(N)) + mild removal (R(M))
5	Low	Mild	Mild	Baseline contact low (C(L)) + mild lockdown (L(M)) + mild removal (R(M))
6	Low	NO	Mild and HQ	Baseline contact low (C(L)) + no lockdown (L(N)) + mild removal (R(M)) + household quarantine (HQ)
7	Low	Mild	Mild and HQ	Baseline contact low (C(L)) + mild lockdown (L(M)) + mild removal (R(M)) + household quarantine (HQ)

a Scenario number.

b Baseline social contact frequency.

c The period with the highest-level alert is identical across scenarios from day 17 to day 32, 16 days in total.

## Results

### COVID-19 transmission networks

Our data of case investigation covered all reported COVID-19 cases between 8 January and 23 February 2020 in Zhejiang, China (Figure 4). Combining all infector–infectee pairs, we thereby got a transmission network of all patients (Figure 4; see *Data Source* for details). We found that the average out-degree for non-singletons (i.e. cases connecting to at least one other case) was 0.72 (95% confidence interval [CI]: 0.68, 0.76), the average shortest path length of clusters was 1.47 (95% CI: 1.26, 1.64), the average betweenness was 0.50 (95% CI: 0.23, 0.82), the average diameter of clusters was 1.30 (95% CI: 1.22, 1.41), and the average size of clusters was 3.52 (95% CI: 3.08, 4.15).

**Figure 3.**
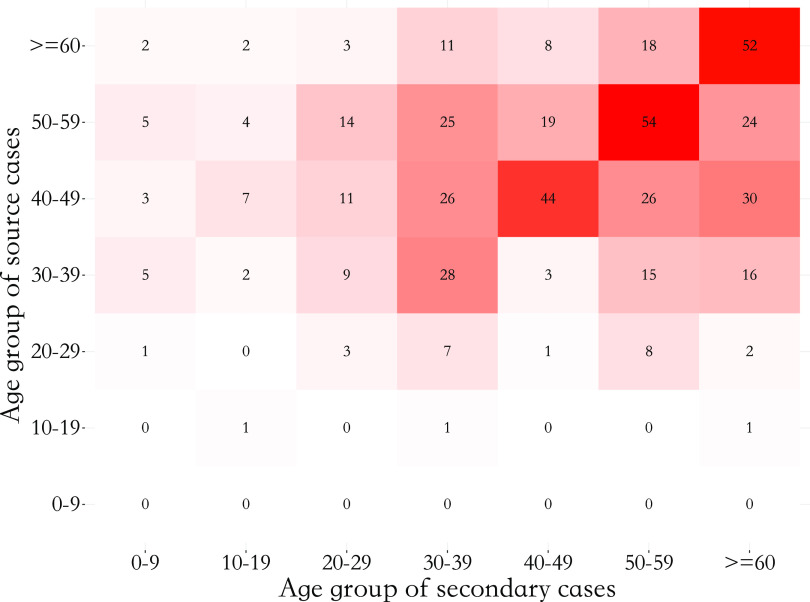
Number of infections between age groups where the depth of colour represents the magnitude of infection number.

**Figure 4.**
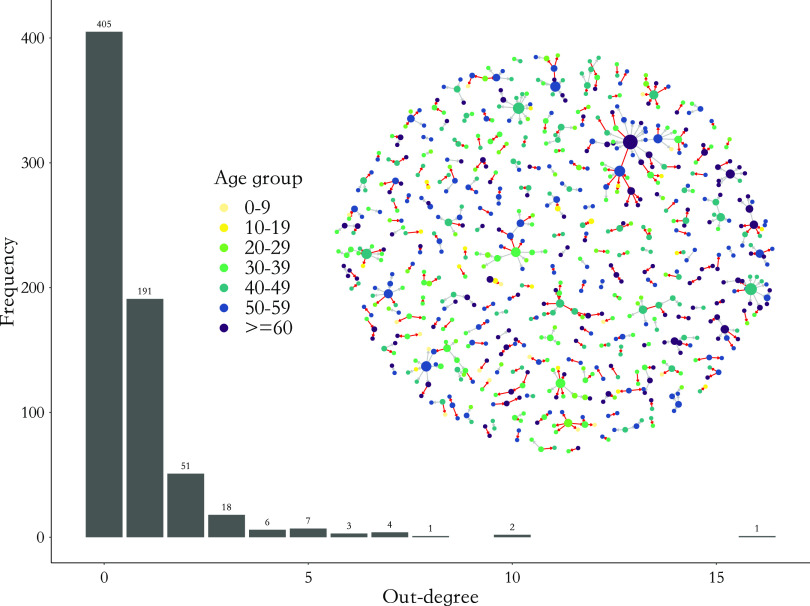
Transmission network for all cases except singletons between 8 January and 23 February 2020 and the histogram of out-degree of each node in the network. The visual network includes nodes and connections from throughout the pandemic in Zhejiang Province and the study time period, including before and after nonpharmaceutical interventions.

There was heterogeneity within the transmission network (Figure 4). Individuals aged between 40 and 49 had the highest mean out-degree (1.10, 95% CI: 0.84, 1.39) and the largest proportion (44.4%, 95% CI: 36.3%, 53.3%) of being the index cases. Individuals 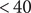 and 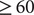 years of age were more likely to be terminal cases (67.4%, 95% CI: 62.5%, 72.0%). Furthermore, the spread between age groups suggested a majority of transmission took place between cases belonging to the same age group (see Figure 3). Household transmission (transmission occurring between family members) accounted for 52.3% (95% CI: 48.1%, 56.8%) of all transmission events. In addition, 54.8% (95% CI: 50.0%, 59.7%) of transmission ended within the household (Supplementary Material, Figure S5).

### The dynamics of graphical structure across time periods

We denoted the time from 8 January to 23 January 2020 as period I, and from 24 January to 23 February as period II (see *Description of network features* for detailed explanation). Sensitivity analysis on the uncertainty of the splitting time point is presented in the Supplementary materials. In the main analysis, there are 776 cases infected in 116 clusters in period I and 573 cases in 78 clusters in period II.

**Figure 5.**
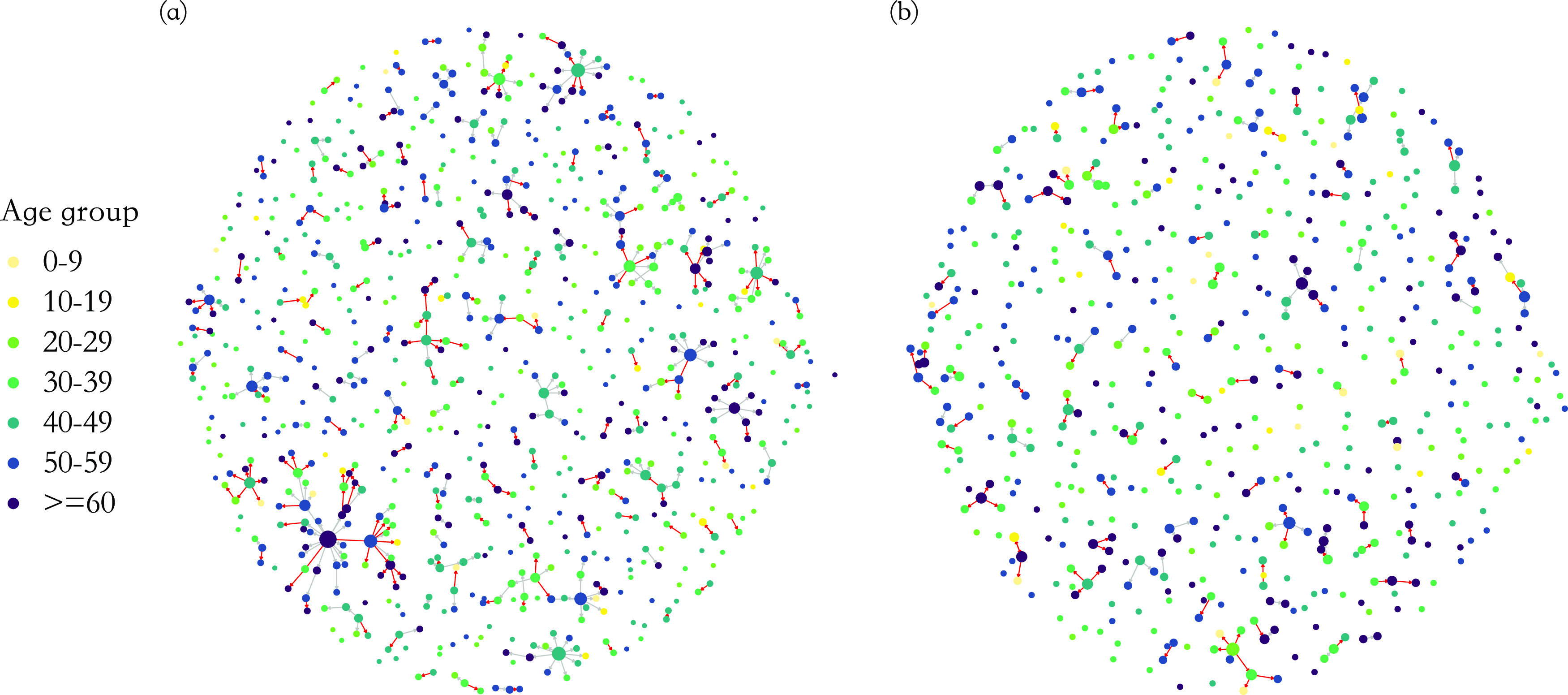
Cases were split into seven age groups, designated by the colours shown in the legend. The size of a node reflects the number of secondary cases it induced (i.e. the magnitude of out-degree). The colour of an edge represents the method of transmission. If transmission occurred within a household, the edge was coloured red; otherwise, the edge was grey; (a) transmission network for cases and clusters originated in period I, before the implementation of large-scale, nonpharmaceutical interventions (23 January); (b) transmission network for cases and clusters originated in period II, after the implementation of large-scale, nonpharmaceutical interventions (24 January).

**Figure 6.**
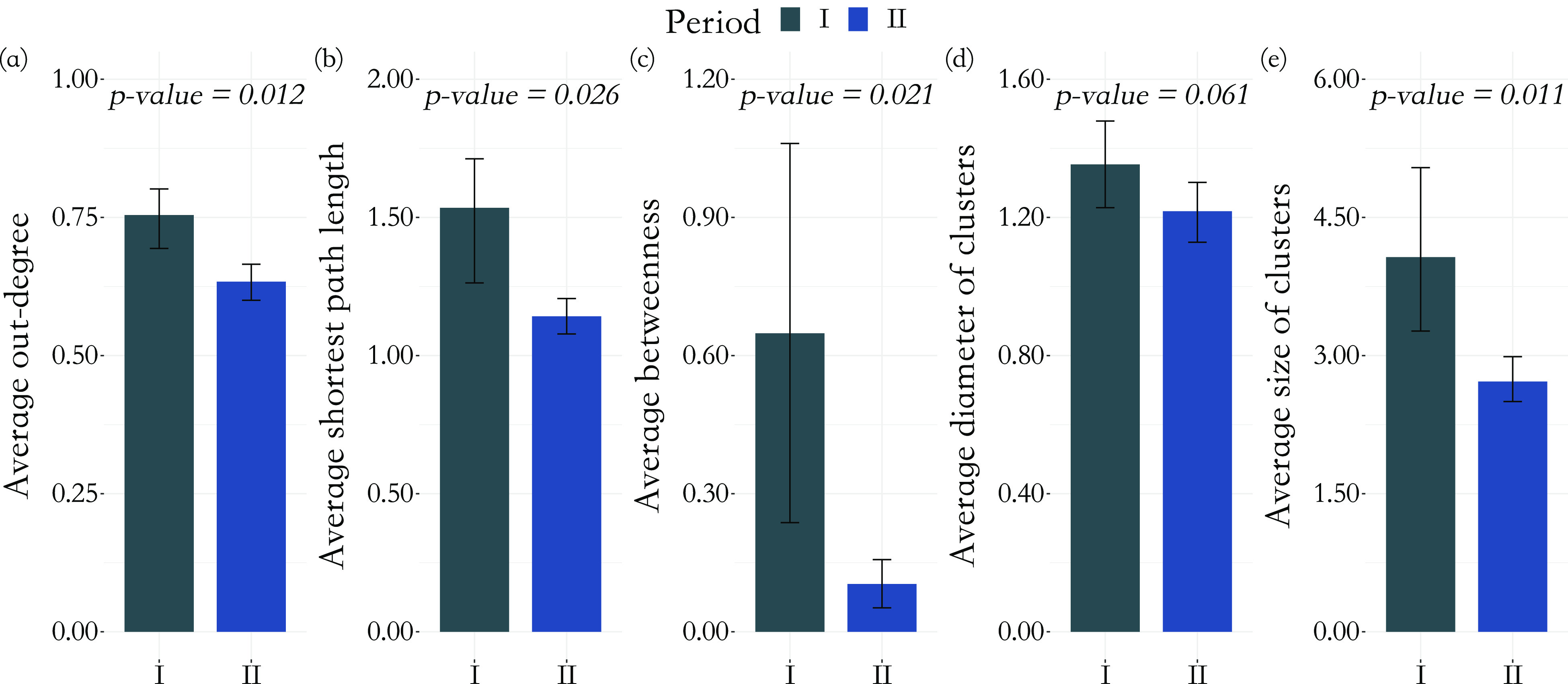
Differences in social network parameters from period I and period II, before and after the implementation of large-scale, nonpharmaceutical interventions. (a) Mean out-degree for non-singletons by period; (b) mean shortest path length by period; (c) average betweenness; (d) mean diameter of clusters by period; and (e) mean size of clusters by period. Student’s 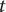 -test was used to compare the means across periods, and the 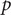 -values were adjusted using the Benjamini–Hochberg procedure. Confidence intervals were estimated from cluster-based bootstrapping.

There were significant changes in several network quantities between period I and period II. The percentage of singleton networks increased from 38.9% (95% CI: 35.2%, 44.0%) to 62.8% (95% CI: 58.4%, 66.6%) 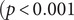 ) between periods I and II (Figure 5), respectively. The increase in singletons indicates the successful implementation of active and effective control measures that disrupted the transmission chains of cases, preventing them from infecting others. In addition, as shown in Figure 6, the average out-degree decreased significantly from 0.75 (95% CI: 0.69, 0.80) to 0.63 (95% CI: 0.60, 0.67), 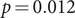 _,_ during the two time periods and so did the average shortest path length (1.53 (95% CI: 1.26, 1.71) to 1.14 (95% CI: 1.08, 1.21), 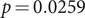 ) and the average betweenness (0.65 (95% CI: 0.24, 1.06) to 0.11 (95% CI: 0.06, 0.16), 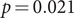 ). Lastly, the average cluster size dropped from 4.05 (95% CI: 3.27, 5.04) in period I to 2.72 (95% CI: 2.50, 2.99) in period II 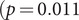 ). Other network quantities remained consistent during the two periods. For example, the mean diameter of clusters (1.35 (95% CI: 1.23, 1.48) to 1.22 (95% CI, 1.13, 1.30), 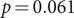 ) remained similar throughout the study time period. Therefore, the virus may spread to a similar generation, but the pattern of transmission had changed to be less extendable (Figure 1 for the hypothetical example). From the histogram of the five graphical measures (Supplementary Material, Figure S7), large outbreaks (clusters with size 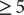 ) were contained and small groups started to dominate in period II. The proportion of superspreader cases (see Methods for details) dropped from 7.4% (95% CI: 5.9%, 8.6%) to 3.3% (95% CI: 1.5%, 5.6%) 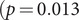 ) from period I to period II.

There was marked dynamic heterogeneity of network structure throughout the province. From period I to period II, the mean out-degree for cases aged between 40 and 59 years largely decreased, while among other age groups, it increased, especially for younger cases aged between 10 to 29 years (Supplementary Material, Figure S6(b)). The proportion of household transmission increased from 47.5% (95% CI: 41.1%, 54.7%) in period I to 65.7% (95% CI: 57.5%, 73.9%) in period II 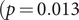 ) (Supplementary Material, Figure S6(c)).

The time from disease onset to confirmation showed a clear declining trend 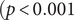 ) from periods I and II (Supplementary Material, Figure S8). The mean number of days for case confirmation was 9.00 (standard error [SE]: 0.21) days in period I compared with 3.94 days (SE: 0.11) 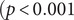 ) in period II. By the end of the outbreak (after 10 February 2020), the mean days to case confirmation were 1.09 days (SE: 0.14).

### The dynamic associations between disease spreading and network structure under NPIs

NPIs can have an impact on both the patterns of interpersonal contacts and the way that disease transmits. Therefore, scenarios under different NPIs could result in rather different infection curves and transmission networks. Using an agent-based susceptible-exposed–infectious-removed (SEIR) model [6, 34] within the framework of an age-dependent transmission and household structure (see *agent-based transmission network model* for details), we first conduct a real-data-based simulation based on parameters estimated from observed data or obtained from the literature (Supplementary Material, Figure S9) that we may refer to as the baseline scenario. The simulated infection curve closely matched the observed data (Supplementary Material, Figure S9). Additionally, the estimated basic reproduction number was around 2.5, which is consistent with the findings of previous studies ranging from 2.6 to 3 [5, 31, 35].

**Figure 7.**
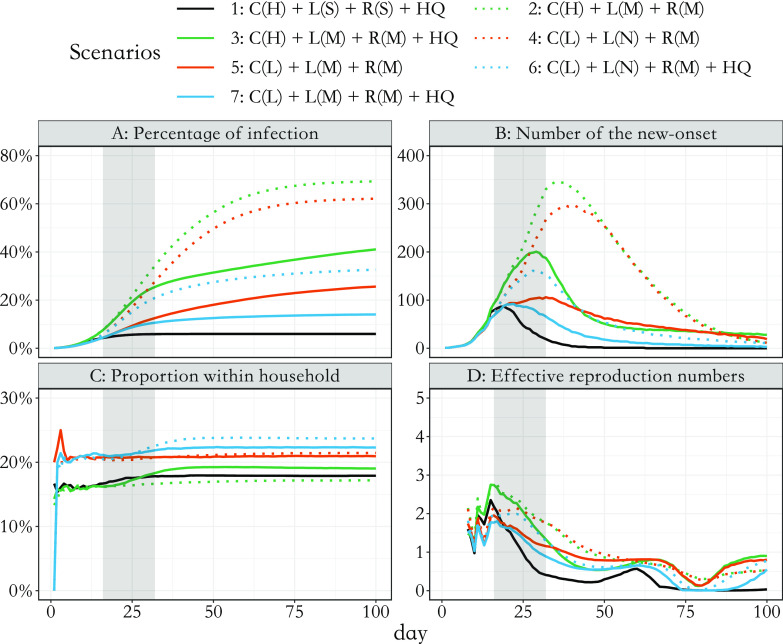
Dynamic change in measures of the outbreak under seven simulated scenarios up to 100 days: (A) percentage of infected people in the total population; (B) the daily number of new cases that show symptoms; (C) accumulative proportion of household transmission; and (D) effective reproduction numbers over weekly sliding windows [37]. Scenario 1 was real-data-based. C(H) and C(L) stand for high and low social contact frequency in the baseline period, respectively. L(S), L(M), and L(N) stand for strict, mild, and no lockdown, respectively. R(S) and R(M) stand for strong and mild active case finding, while HQ stands for active household quarantine policy. Shaded areas (from days 17 to 32) represent the period with the highest-level alert to the pandemic. The resumption of social contact rate begins from day 33 to day 63.

**Figure 8.**
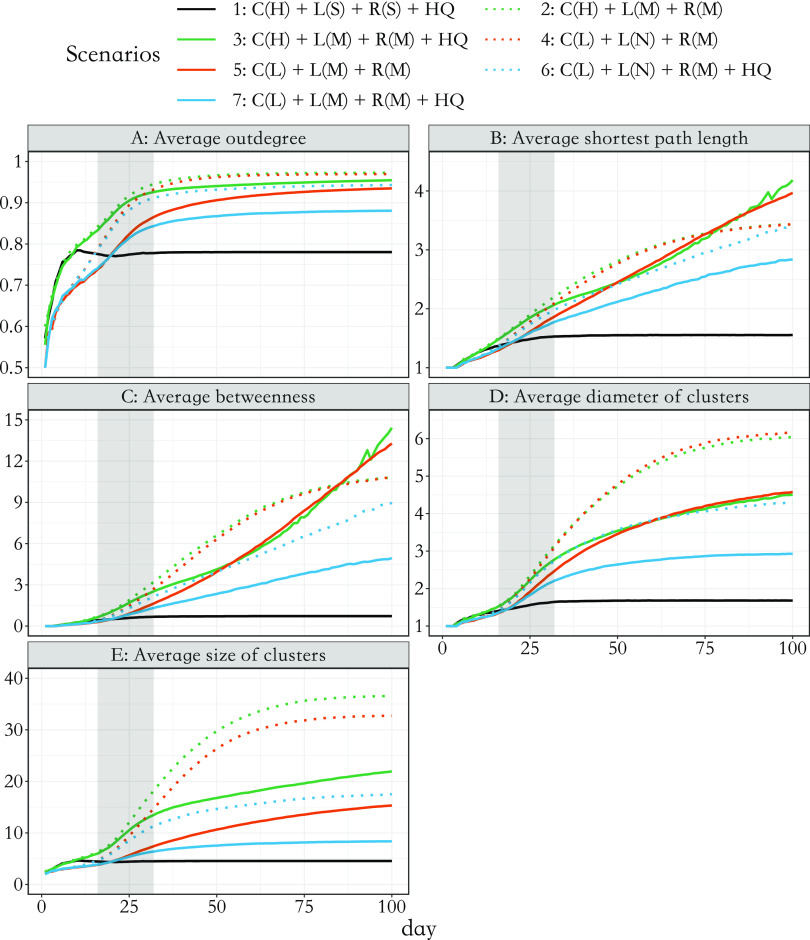
(A) average out-degree for non-singletons in the network; (B) average shortest path length; (C) average betweenness; (D) average diameter of clusters; and (E) average size of clusters; each of them on a specific day is calculated on the network up to that day. Other components in this figure remain the same as in Figure 7.

Based on the baseline scenario that closely simulates the situation in Zhejiang, we further explored epidemic development and its underlying graphical structure under various intervention scenarios (Table 1). We varied baseline (pre-outbreak) social contact frequency to better represent locations with less population density. The first three scenarios represent an area with a high baseline contact frequency (C(H)), such as Shanghai, while the rest represent a lower contact frequency (C(L)) for a sparsely populated area. The impact of social distancing, active case finding [36], and household quarantine on subsequent outbreaks and graphical measures were also estimated. Results are presented in Figures 7 and 8 in which lines with the same colours or same line styles (e.g. solid versus dotted green line) represent scenarios that share a similar setting (e.g. solid lines typically have a stronger NPI compared with dotted lines). In particular, scenario 3 (in solid green) adopted the household quarantine policy compared with scenario 2 (in dotted green); the intensity of social distancing raised to a mild level in scenario 5 (in solid red) versus scenario 4 (in dotted red) and in scenario 7 (in solid blue) versus scenario 6 (in dotted blue). The simulation results are separated into two parts, one for the development of outbreak processes (Figure 7) and the other for the network measures that describe the expansion of the simulated transmission networks (Figure 8). The corresponding uncertainty assessments, represented by confidence bands, can be found in the Supplementary materials, specifically in Figures S10 and S11 in the Supplementary Material. From simulations, we found that the dynamic change in network structural attributes is related to the development of the outbreak and provides additional insights apart from the general infection curve.

Although average out-degree is an essential measure to describe the ability of reproduction, it is not sensitive even to large differences in population density and relatively strong control policies (Figure 8A); there is only a relative 10% difference between scenarios except the real-data-based case. Moreover, compared to the infection curve (Figure 7A) and other attributes (Figure 8B-E), the increasing rate (i.e. gradient) of average out-degree stabilises faster and reaches a plateau much earlier. Hence, one could have falsely concluded that the outbreak processes start to be suppressed if only observing the dynamic trend of average out-degree. On the contrary, the average size of clusters has a similar pattern as that seen in the corresponding percentage of infection (Figure 7A versus Figure 8E). It is also most sensitive to control policies. Furthermore, the average shortest path length, average betweenness, average diameter, and size of clusters (Figure 8B-E) can suggest the coming of the turning point of the outbreak; when they start to stabilise, the number of new onsets (Figure 7B) also reaches its turning point and the effective reproduction numbers (Figure 7D) over weekly sliding windows drop below 1. However, if they still increase with a non-decreasing gradient (such as in scenarios 3 and 5, solid red line in Figure 8B-E) the number of the new onsets could stay flat and never encounter a turning point.

For scenarios with insufficient interventions (scenarios 2 and 4), they ended up with roughly 60–70% of infections among the total population and the transmission network stopped growing quickly due to herd immunity. For scenarios with mild interventions (scenarios 3 and 5), they began with a much slower outbreak. However, following the resurgence in social contact from day 33 to day 63, the growth of infections also resumed and reached a plateau. From visual inspections, the average shortest path length, average diameter, and size of clusters followed a linearly increasing trend and the gradient of average betweenness also increased (Figure 8B-E). These features suggest that the ability of network expansion has not been suppressed. For future containment of the virus, tailored and more stringent controlling policies may be needed.

## Discussion

Population-level COVID-19 social networks are poorly studied but critical to understand COVID-19 transmission dynamics. We observed heterogeneities in the transmission network of a COVID-19 outbreak and found that its dynamics were associated with social characteristics. Control interventions can lead to changes in network structures, which in turn affect their effectiveness.

In this study, we presented the dynamic characteristics of COVID-19 patient social networks among all cases diagnosed in Zhejiang Province, China. Before the COVID-19 pandemic [7, 38, 39], previous studies using patient networks have thus far been relatively limited to HIV and tuberculosis [40–42]. Interventions that successfully broke down infection clusters into smaller ones better mitigated the transmission chains but may alter the network structures by reducing the risk for some individuals while increasing it for others, such as household contacts. Upon re-simulating patient networks using an age-dependent agent-based network susceptible-exposed–infectious-removed (SEIR) model, our results demonstrated an important contribution of network structure associated with population-level COVID-19 transmission and the dynamics of specific interventions. The methodology used is applicable to other locations and future pandemics to understand the influence of topological characteristics and the influence of tailored intervention strategies.

We measured five commonly used network features, including out-degree, shortest path length, betweenness centrality, the diameter of clusters, and cluster size. Overall, the COVID-19 outbreak in Zhejiang Province was moderate, with an average of 1.31 spreading generations and 3.52 infection cluster sizes. Distinct from other descriptions [7, 18, 43], large clusters in the province were uncommon. After the widespread implementation of NPIs, the network in period II was more fragmented and with significantly reduced average out-degree, average shortest path length, betweenness centrality, and cluster size, indicating that social distancing decreased the capability of viral transmission. However, there was a limited impact on reducing generations produced from a single index case. With fewer network branches produced by a single case in period II (largely due to NPIs), suppression of the population-level pandemic was considered manageable.

Our results also suggest that social distancing alone is unlikely to decrease social connections equally. The average number of secondary cases produced by younger populations was less impacted between period I and period II compared with older participants (specifically middle-aged individuals). This suggests that age-dependent transmissibility and susceptibility were present in our analysis and policies restricting social contact introduced heterogeneous effects on the population. Importantly, the proportion of household transmissions increased in period II, suggesting additional efforts were needed for household member protection.

Through simulations in seven distinct epidemiological scenarios, we found that network characteristics contribute useful insights into certain aspects of the pandemic. For example, the average size of clusters had an implication on the total scale of the outbreak, while the average diameter of clusters may suggest the existing expansion capability of the pandemic and the shortest path length may unveil the potential of further expansion. Aligned with the results observed in periods I and II, we found that out-degree was essential to the size of the outbreak but not sensitive to implemented interventions and stabilised much sooner than other attributes. Therefore, it may lead to misjudgement of the further development of the outbreak by only observing the trend of out-degree. The model also confirmed that relaxing the intensities of physical control and case finding could have led to severe uplift of the scale of an outbreak and slower convergence of graphical characteristics regardless of population density. Active case finding (case isolation and household quarantine) reduced transmission by suppressing new branches and blocking edges in the network.

Our study has limitations. Transmission directions within networks were mostly based on symptom onset. Although this methodology has been widely used in other studies [8, 38, 44], there is potential for misclassification, leading to the reversal of several transmission pathways. Also, some singletons could be attributed to missed epidemiological investigations. Network recovery approaches for transmission networks are essential to restore lost epidemiological connections. With more complete transmission networks, the analysis will yield deeper and more accurate insights into the dynamics of disease spread. In addition, we were unable to rebuild social networks of asymptomatic cases due to a lack of symptom onset among these patients. Our study may not accurately represent transmission dynamics in communities with higher levels of transmission, such as those exposed to novel variants [39, 45]. However, a description of the social network structure and its influence on the broader epidemic, the primary aim of this analysis, may be useful for understanding dynamics in these settings. Lastly, in our data, only 9% of the confirmed cases were marked as asymptomatic, and knowledge of asymptomatic cases was also limited. Thus, it may not be sufficient to study the characteristics of asymptomatic cases. Furthermore, in early 2020, transmission network data of asymptomatic cases were also rarely reported. Evidence suggested that their transmissibility may be lower than in symptomatic cases. The lower transmissibility but ability of escaping case detection caused difficulties to estimate the impact of NPIs accurately. Our study mainly focused on symptomatic cases, but if the asymptomatic transmission burden was indeed higher, we expect that the scenario comparison results still hold, but the scale of an outbreak would be bigger. Vaccination was also unavailable during the time period of data collection, and the data exploration and simulation in this article may not reliably describe transmission patterns among asymptomatic or vaccinated individuals. By comparing the epidemiological investigation data on novel variants and COVID-19 transmission after vaccination, we can uncover the graphical transmission patterns of novel variants and the effectiveness of vaccines in network interventions.

In summary, we describe and report topological structures and dynamic changes in COVID-19 transmission networks through a population-based, surveillance system of all diagnosed cases in Zhejiang Province, China. By integrating several surveillance datasets, the results from our agent-based network model suggest that structural characteristics are dynamically related to COVID-19 spread at the population level. Network characteristics are important to the overall COVID-19 pandemic and have clear implications for the impact of interventions.

## Supporting information

He et al. supplementary material He et al. supplementary material

## Data Availability

Code for data investigations and simulations is publicly available on https://github.com/howanchung/COVID19-network.

## Supplementary material

For supplementary material accompanying this paper visit http://doi.org/10.1017/S0950268823001292. click here to view supplementary material
