# Supplementary material for: Social mixing and network characteristics of COVID-19 patients before and after widespread interventions: A population-based study
Source: Epidemiol Infect. 2023 Aug 14;151:e141. doi: 10.1017/S0950268823001292 (PMC10540215; doi:10.1017/S0950268823001292)
Supplement: He et al. supplementary material [file S0950268823001292sup001.pdf]

Supplementary Information for: Social Mixing and Network Characteristics of COVID-19 Patients Before and After Widespread Interventions: A Population-based Study

Yuncong He[1†], Leonardo Martinez[2†], Yang Ge[3†], Yan Feng[4†], Yewen Chen[5†], Jianbin Tan[1], Adrianna Westbrook[5], Changwei Li[6], Wei Cheng[4], Feng Ling[4], Huimin Cheng[7], Shushan Wu[7], Wenxuan Zhong[7], Andreas Handel[5], Hui Huang[8*], Jimin Sun[4*] and Ye Shen[5*]

[1]School of Mathematics, Sun Yat-sen University, Guangzhou, 510275, Guangdong, China.
[2]Department of Epidemiology, School of Public Health, Boston University, Boston, 02118, Massachusetts, United States.
[3]School of Health Professions, University of Southern Mississippi, Hattiesburg, 39402, Mississippi, United States.
[4]Zhejiang Provincial Center for Disease Control and Prevention, Hangzhou, 310051, Zhejiang, China.
[5]Department of Epidemiology and Biostatistics, College of Public Health, University of Georgia, Athens, 30602, Georgia, United States.
[6]Department of Epidemiology, Tulane University School of Public Health and Tropical Medicine, New Orleans, 70112, Louisiana, United States.
[7]Department of Statistics, University of Georgia, New Athens, 30602, Georgia, United States.
[8]Center for Applied Statistics and School of Statistics, Renmin University of China, Beijing, 100872, China.

*Corresponding author(s). E-mail(s):
huangh89@mail.sysu.edu.cn; jmsun@cdc.zj.cn; yeshen@uga.edu;
†These authors contributed equally to this work.

Data souce

We collected individual-level data on 1349 SARS-CoV-2 infections during the major outbreak in Zhejiang province, China, from January 8th to February 23rd, 2020 [1, 2]. The data contain comprehensive information of each case, such as their demographical information and epidemiological linkage to others identified through contact tracing efforts. The resulted infector-infectee transmission pairs thereby inherently contain the nature of a directed transmission network. Precisely, started from each indexed case, edges consecutively direct out to the secondary infections, which ultimately form a cluster or a "component" in the terminology of network science. The infector-infectee transmission pairs form a transmission network combining such clusters. The nodes and edges inside are unlikely to be homogeneous, and the topological characteristics of the networks across periods are also distinct. To better understand the patterns and drivers of such heterogeneity, we analyze four fundamental graphical measures introduced below, both statically and dynamically.

The personal information for each case includes age, gender, guardians (if applicable), household information, household and occupational location, the severity of infection, potential exposures, travel history to other areas, date of symptom onset, and date of laboratory-confirmation. Potential exposures are recorded in detailed texts where we extracted names and types of exposures. All epidemiological information and laboratory confirmation were collected by specialists in provincial or municipal CDC or hospitals in Zhejiang. Extra efforts were conducted to correct typographical errors (such as names in the same pronunciation) and explore additional information such as family relation that is missed in raw records.

Methods

Notation of network and definitions of network characteristics

We use a 2-tuple (N, g) to represent a network object, where N is the set of indexes of nodes and the adjacency matrix g is a real-valued $n \times n$ matrix, in which entry g_{ij} represents the relation between i and j. In a transmission network with specified transmission directions, $g_{ij} = 1$ means that i is the source case of j and j is the secondary case of i. The out-degree of a node is the number of edges directed out from it and the in-degree of a node is the number of edges that ends with it. We let d_i^+ and d_i^- denote the out-degree

and in-degree of the ith node, respectively. Thus if $d_i^+ > 0$ and $d_i^- = 0$, the ith node is an indexed case which is the origin of a cluster. On the other hand, if $d_i^+ = 0$ and $d_i^- > 0$, the ith node is a terminal case that induces no other secondary case. If both $d_i^+ = 0$ and $d_i^- = 0$, the ith node does not belong to any cluster, and it is marked as a singleton in the network. We let S denote the set of singletons. The reasons for a node becoming a singleton are twofold. For one thing, it was an imported case and did not infect anybody. For another, the transmission linkage of it was inexplicit, and epidemiologists were not able to identify neither the source case nor secondary cases of it. For example, there was a large outbreak within a prison, but we were unable to identify an explicit transmission chain, and thus, most of the involved cases were considered singletons. In calculating network characteristics, we neglect the existence of singletons inside; i.e., we focus on the sub-network $N \setminus S$.

Throughout this article, we mainly consider four basic graphical measures of the transmission network: 1) Average out-degree, 2) average shortest path length, 3) diameter of clusters, and 4) sizes of clusters, which would characterize the number of secondary cases, the cohesion of the transmission occurrence, the generations of the epidemic spread and the developed size for one clustered epidemic event, respectively. The average out-degree is the sum of out-degree for non-singleton cases divided by the total number of them, that is $\sum_{j \in N \setminus S} d_j^+ / |N \setminus S|$, where $|\cdot|$ denotes the cardinality of a set. For the average shortest path length (ASPL), we firstly define that $\mathrm{path}(i,j) = 1$ if there exists a directed path beginning from the ith and ending in the jth nodes, otherwise $\mathrm{path}(i,j) = 0$. Among all paths, the distance between the ith and the jth nodes, $\mathrm{dist}(i,j)$ is defined as the length of their shortest path (i.e., geodesic). If $\mathrm{path}(i,j) = 0$, we assume the distance between them is 0. The average shortest path length is defined as [3]

$$\mathrm{ASPL} = \frac{\sum_{i,j \in N, i \neq j} \mathrm{dist}(i,j)}{\sum_{i,j \in N, i \neq j} \mathrm{path}(i,j)} \tag{S1}$$

Moreover, betweenness centrality of node v is defined as

$$C_B(v) = \sum_{i,j : i \neq j \neq v} \frac{g_{ivj}}{g_{ij}} \tag{S2}$$

where g_{ij} is the total number of shortest paths from node i to node j and g_{ivj} is the total number of shortest paths from node i to node j via node v. Therefore, $C_B(v)$ quantifies the information transportation that passes through node v. Furthermore, information is originated from instead of transporting through the source node of a tree-shaped network, the betweenness centrality of it is always zero. Similarly, the betweenness centrality of a terminal node inside a tree-shaped network is also zero. Therefore, in a transmission network (composed of tree-shaped sub-networks), only the intermediate nodes between the source and terminal nodes contribute to the measure of betweenness.

The diameter of a connected transmission network is the maximum distance within it; i.e., the distance from the indexed case to the farthest terminal case. In the context of epidemiology, the diameter of a transmission network is the maximum generation of the virus spread within it. Since the whole transmission network can be decomposed into a collection of clusters or components, we calculate the average diameter of clusters by averaging the diameter of each cluster. Lastly, the size of a sub-network is the number of nodes within it. Thus, the average size of clusters is calculated by averaging the sizes of clusters.

Agent-based transmission network model for simulations

A detailed description of how we build an agent-based transmission network model is presented in the main text. Here we provide supplementary materials on its settings. Key parameters for the outbreak reconstruction are summarized in Table S1. As described in the main text, we first build a social network considering the household, geographical, and random connections between people. Specifically, we utilize our observational data to give realistic settings for parameters such as household size and family-based age distribution. In addition, we construct a social connection network consistent with the age-specific contact rates matrix explored in detail by Zhang et al. [4] and assign weights in different connections.

In terms of the transmission processes, we consider both pre-symptomatic infectiousness and post-symptomatic viral shedding. More precisely, patients are able to transmit COVID-19 before showing symptoms [5] (pre-symptomatic infectiousness) and will lose infectiousness afterwards due to insufficient viral loading [5, 6]. Thus, we assume five compartments in our model: susceptible, exposed, pre-symptomatic infectious, post-symptomatic infectious and removed state. Every node is initially susceptible. After exposure to known infectious cases, a node has a probability ($1-(1-\beta)^n$ in the main-text) to be infected and will then be transferred to the exposed state. In the exposed state, cases are non-infectious. After a period of time (we assume it as a proportion of the incubation period, the duration from being infected to symptom onset), cases will be transferred to the pre-symptomatic infectious state. Afterwards, cases will show symptoms and move to the post-symptomatic infectious state. As long as they develop symptoms, they will be assigned a removal period, the duration between symptom onset and isolation. After a removal period, cases will be quarantined and no longer participate in the transmission processes, i.e., move to the removed state. Note that in very early stage of the outbreak, the speed of case finding is relatively slow. Therefore, the removal period can be longer than the post-symptomatic infectious period as cases could be physically free while already losing their infectiousness capabilities. Settings for those periods as well as age-dependent heterogeneity are in accordance with some previous studies [5–7]. For the removal of infected cases, we set the removal period based on our observational data (see Fig. S8 (a)). Moreover, we incorporated a dynamic change of contact pattern through the pre-outbreak, lockdown, and resumption phases based on previously reported

contact matrices observed during the pre-outbreak and outbreak periods [4] (see *Construction of daily age-specific contact matrix*, Fig. S2 and Fig. S3).

Construction of daily age-specific contact matrix

According to Zhang et al. [4], we can get the age-specific contact matrix for both baseline period and outbreak period, denoted by c_{base} and c_{outbreak}. However, neither the intermediate state in between nor the contact pattern in the post-lockdown period was observed. Therefore, we assume that it decrease as a time-dependent function following Tan et al. [8] (Fig. S3). At the beginning, we assume the contact rate declined in a very small scale (ϵ) before January 10th (3 days from January 8th) and started to drop afterwards. Zhejiang provincial government upgraded its infectious disease alert category to the highest level on January 23rd, 2020, we assumed that the social contact frequency dropped to the lowest level afterwards. The provincial government started the reopening on February 10th, 33 days from January 8th, after which the social contact frequency increased. Because COVID-19 cases were still being reported sporadically, we assumed the social contact frequency, in the following one month, equal to an average of the contact levels in the baseline period and the outbreak period. Briefly, let $c^{(d)}$ be the contact matrix in the d day. Then $c^{(0)} = c_{\text{base}}$, $c^{(d)} = c_{\text{outbreak}}$ for $16 \leq d \leq 32$ and $c^{(d)} = (c_{\text{base}} + c_{\text{outbreak}})/2$ for $d \geq 63$. We denote the average contact number of the ith age group to the jth age group at time d as $c_{ij}^{(d)}$. For $1 \leq d \leq 16$, the monotonic decline function followed a logistic curve given by the following equation:

$$c_{ij}^{(d)} = c_{ij}^{(0)} \left(\frac{1 - \eta_{ij}}{1 + \exp(\lambda_m (d - t_0 - m/2))} + \eta_{ij} \right) \tag{S3}$$

Here, if λ_m is chosen as $2 \log(\epsilon/(1-\epsilon))/m$ and ϵ is sufficiently small (e.g. $\varepsilon = 0.01$), m could be viewed as the duration of the decreasing process [8] (as illustrated in Fig. S2). $\eta_{ij} = c_{ij}^{(16)}/c_{ij}^{(0)}$ is the percentage of decrease. As discussed above, we set $m = 13$, $t_0 = 3$. On the other hand, in terms of the resumption process, for $32 \leq d \leq 62$, we invert the decreasing process by:

$$c_{ij}^{(d)} = c_{ij}^{(63)} \left(\frac{1 - \gamma_{ij}}{1 + \exp(\lambda_m (62 - d - m/2))} + \gamma_{ij} \right) \tag{S4}$$

where $m = 30$ with λ_m as defined above and $\gamma_{ij} = c_{ij}^{(32)}/c_{ij}^{(62)}$. In a nutshell, we can get a series of contact matrices for every day d ($c^{(d)}$) presented in Fig. S3.

Exploration and supporting results

Graphical characteristics of the observed transmission data

We collected data on 1349 confirmed SARS-CoV-2 infections identified in Zhejiang Province as well as their baseline information and epidemiological

tracing notes. From information collected through contact tracing, we partially recovered the infector-infectee transmission chains between cases. If one case had more than one potential source of infection, we sampled only one. Sensitivity results are presented in *variation of sampling a source case*. Thereafter, a transmission network can be constructed by combining all transmission pairs. We then computed four basic graphical measures to assess the transmission network quantitatively: 1) Average out-degree, 2) average shortest path length, 3) diameter of clusters, and 4) sizes of clusters. Among them, there exists heterogeneity related to demographical factors such as age and household transmission. Relatively older adult cases accounted for more significant contributions than younger adult and adolescent cases in the transmission processes (top-right corner in Fig. S4 (a)). Notably, the average out-degree (i.e., number of secondary infections) induced by cases aged between 40 and 59 is considerably higher than that induced by cases from other age groups (Fig. S4 (b)). Furthermore, those aged between 40 and 59 were more frequently identified as the indexed cases in the transmission network, while cases from other age groups were substantially more likely to be positioned as the terminal cases. In terms of household transmission, 54.5% of the terminal transmission occurred within the household, while household transmission only accounted for 39.3% of non-terminal transmission.

Dynamic epidemiological and graphical characteristics of the transmission network

Across periods before and during the outbreak (that is period I and period II), there existed a quantity of change both on epidemiological and graphical aspects. First, the average removal period (the duration from symptom onset to isolation, which reflects the speed of case finding) was decreasing over time, starting from nearly 20 days and ending with virtually zero (Fig. S8 (a)). In aggregation, the average removal period in period I was 6.41 days, while in period II, it was 4.18 days. Moreover, the curve of imported cases over time peaked at the declaration of lock-down, with most of them from period I (Fig. S8 (b)). On a graphical aspect, the secondary infection induced by cases of all ages showed a clear distinction between periods (Fig. S6 (b)). Cases aged between 40 and 59, the age groups with the largest average out-degree in period I, encountered a considerable decrease in this quantity in period II. However, for all other ages, the secondary infection increased. Their wax and wane jointly resulted in a relatively homogeneous distribution in average out-degree across age groups in period II. Besides, the heterogeneity of cases' location in the network related to their age also changed by periods (Fig. S6 (a)). Except in those below 20, cases belonging to other age groups had an increasing proportion as an indexed case between periods I and II. Conversely, the proportion as a terminal case dropped in nearly all age groups. Household transmission often resulted in terminal transmission during both periods, but that proportion considerably rose from period I to period II. In addition, from period I to II, some graphical measures such as average out-degree, average

shorted path length, and the average size of clusters encountered noticeable recession. Both the large-spreading events (each with an out-degree at least 3) and large clusters (with a size of at least 5) became less common in period II. Most of the shortest path length between cases concentrated below 2, also suggesting that clusters became much more cohesive around their indexed cases and less forked.

Reconstruction of the transmission network

Using the agent-based transmission network model described in the main text as well as *Agent-based transmission network model for simulations* under realistic settings given in Table S1, we reconstructed a transmission network for the outbreak in Zhejiang from January 8th to February 23rd. From Fig. S9, the daily number of the new-onset cases in reconstruction fitted well to the observed one.

Sensitivity analysis on the split-point of time periods

January 20th as the split-point

We chose the time before January 20th as the first period and the time after January 21st as the second period and repeat the analysis on the main text. The results show that some network attributes significantly changed across periods. In details, the proportion of singletons significantly increased from 32.4% (95% CI: 27.6%, 37.4%) to 58.8% (95% CI: 55.4%, 62.7%) ($p < 0.001$); average out-degree from 0.77 (95% CI: 0.69, 0.83) to 0.66 (95% CI: 0.62, 0.70) ($p = 0.012$); average shortest path length from 1.63 (95% CI: 1.25, 1.77) to 1.17 (95% CI: 1.10, 1.21) ($p = 0.012$); average diameter of clusters from 1.41 (95% CI: 1.23, 1.55) to 1.23 (95% CI: 1.15, 1.30) ($p = 0.042$); average size of clusters from 4.41 (95% CI: 3.19, 5.84) to 2.97 (95% CI: 2.62, 3.32) ($p = 0.0122$); the proportion of household transmission from 45.7% (95% CI: 38.6%, 54.0%) to 59.4% (95% CI:52.0%, 67.7%) ($p = 0.042$), average removal period from 10.29 days (SE: 0.28) to 4.48 days (SE: 0.11) ($p < 0.001$); the number of clusters increased from 75 to 119. Only one attribute's change was statistically insignificant, the proportion of super-spreaders from 7.7% (95% CI: 5.1%, 9.3%) to 4.8% (95% CI: 3.0%, 6.4%) ($p = 0.065$).

Compared to the result on the main text using January 23 as the split-point, on a significance level of 0.05, there are two statistically different results: the decrease of proportion of super-spreaders became insignificant and the decrease of average diameter of clusters become significant. This is because after re-allocating the time between January 21st and January 23rd into period II, the number of super-spreading events in period II and the average diameter of clusters in period I increased significantly.

February 1st as the split-point

We chose the time before February 1st as the first period and the time after February 2nd as the second period and repeat the analysis on the main text. The results show that nearly all network attributes insignificantly changed across periods. In details, the proportion of singletons significantly increased from 45.6% (95% CI: 42.4%, 48.6%) to 85.5% (95% CI: 79.1%, 91.1%) ($p < 0.001$); the number of clusters largely dropped from 186 to 8. The change of other attributes was statistically insignificant: the proportion of super-spreaders from 6.3% (95% CI: 4.8%, 7.4%) to 0.0% (95% CI: 0.0%, 0.0%) ($p = 0.3453$); average out-degree from 0.72 (95% CI: 0.67, 0.77) to 0.56 (95% CI: 0.50, 0.61) ($p = 0.158$); average shorted path length from 1.47 (95% CI: 1.25, 1.65) to 1.00 (95% CI: 1.00, 1.00) ($p = 0.137$); average betweenness centrality from 0.52 (95% CI: 0.22, 0.89) to 0.00 (95% CI: 0.00, 0.00) ($p = 0.179$); average diameter of clusters from 1.32 (95% CI: 1.23, 1.42) to 1.00 (95% CI: 1.00, 1.00) ($p = 0.137$); average size of clusters from 3.59 (95% CI: 3.05, 4.34) to 2.25 (95% CI: 2.00, 2.57) ($p = 0.147$); the proportion of household transmission from 52.0% (95% CI: 44.2%, 60.0%) to 60.0% (95% CI: 36.4%, 94.7%) ($p = 0.749$). Average removal period dropped significantly from 6.63 days (SE: 0.13) to 2.06 days (SE: 0.13) ($p < 0.001$).

Compared to the result on the main text using January 23 as the split-point, on a significance level of 0.05, nearly all statistically significant results changed to as insignificant. In period II (after February 1st), the total number of cases and clusters both dropped significantly, which could have caused reduced statistical powers.

Variation of sampling a source case

We also conduct a sensitivity analysis on assessing the variation of sampling a source case. There are 43 cases with more than one potential source cases. For such cases, we randomly sample one ”true” parent among all potential source cases and repeat the analysis on the main text.

Among 100 repetitions, results on comparison across periods in the main text are consistent except the proportion of superspreaders, average shortest path length and average diameter of clusters. 29% of the comparisons on the proportion of superspreaders, 19% on the average shortest path length and 65% on the average diameter of clusters vary from the results in the main text.

Supporting data

Age distribution in Zhejiang

According to the census data on Zhejiang [9], the age distribution on Zhejiang on the year 2020 is as follows:

Distribution of family size

According to the observed data, the distribution of family size is illustrated in Fig. S12.

Age distribution by different family size

According to the observed data, the age distribution on a sample of families of different size is presented in Table S2, in which number represents age year.

Table S1: Key parameters for the network simulation

Process	Type	Description	Value or distribution
Social Network	Network size	The number of nodes	20,000
	Household Connections	The average size of groups	2.94
		Weight in household connections	Base: 3.716; Outbreak: 5.041
	Geographical Connections	Number of geographical connections	If a node is in ith age group, the number of geographical connections of it is $2n_i$, where n_i is the average number of contacts per day of the ith age group, i.e., the sum of ith row in base contact matrix in Shanghai [4]
		Number of geographical connections to people in each group	Multinomial distribution with the total number being $2n_i$ and the probability being the normalized ith row of base contact matrix in Shanghai.
		Weight in geographical connections	0.5
	Random Connections	The mean number of random connections per person	1
		Weight in random connections	0.5
Transmission processes	Period	Incubation period	Log-normal$(4.2, 1.9)$ (i.e. mean and standard deviations of the logarithms are 4.2 and 1.9 respectively) [5, 10]
		Duration on exposed state: time from being infected to being infectious	Incubation period / Log-normal(0.04, 0.59)
		Pre-symptomatic infectious period	Incubation period − the duration on exposed state
		Post-symptomatic infectious period	Gamma distribution with parameter 5.2 and 8
		Removal period	Weibull distribution with mean μ_t on day t and scale $\mu_t/\Gamma(1.2)$ is assigned according to Fig. S8 (a)
	Infection rate	Infection rate per contact (β)	Peak at 5% [11] (see Table S3)
	Susceptibility	Susceptibility in each age group	See Fig. S1 [12]
	Contact number	Daily contact number to each age group	Contact matrix in each day calculated from contact matrix in Shanghai before and during the outbreak, see Fig. S3
		The pattern of decreasing and resuming	See Fig. S3
	Imported cases	Total number of imported cases	445
		Import day	See Fig. S8 (b)

Table S2: Age distribution by different family size

Family size	Age distribution
2	(13, 40), (31, 32), (58, 63), (26, 49), (33, 63), (57, 61), (42, 45), (71, 74), (37, 63), (55, 72), (59, 59), (7, 36), (26, 52), (30, 35), (23, 57), (45, 47), (64, 88), (50, 53), (60, 62), (34, 35), (36, 49), (36, 40), (53, 53), (29, 58), (54, 56), (29, 51), (37, 40), (62, 71), (41, 42), (54, 55), (47, 48), (45, 69), (43, 68)
3	(32, 37, 60), (4, 10, 33), (31, 62, 85), (51, 59, 63), (7, 30, 31), (29, 37, 63), (62, 64, 66), (6, 36, 53), (0, 32, 56), (1, 34, 68), (39, 41, 43), (9, 12, 62), (36, 63, 66), (20, 44, 50), (12, 45, 45), (34, 57, 62), (51, 83, 90), (35, 56, 59), (51, 77, 79), (20, 45, 46), (38, 64, 67), (38, 40, 63), (12, 40, 43)
4	(24, 34, 41, 60), (10, 19, 41, 44), (39, 39, 66, 67), (29, 48, 51, 70), (29, 30, 31, 32), (35, 38, 67, 70), (0, 29, 51, 54), (18, 20, 42, 67), (26, 51, 80, 85), (43, 47, 70, 72), (10, 39, 39, 66), (28, 49, 51, 53), (23, 40, 49, 71)
5	(57, 58, 62, 65, 90), (11, 35, 37, 67, 69), (10, 37, 37, 38, 64), (19, 37, 44, 47, 86), (47, 48, 50, 51, 70), (31, 56, 57, 59, 85), (37, 41, 46, 47, 70), (38, 61, 64, 67, 69)
6	(13, 44, 46, 66, 74, 81), (1, 31, 32, 56, 57, 74), (22, 35, 47, 59, 60, 73)
8	(1, 22, 24, 32, 47, 56, 72, 73), (3, 6, 29, 31, 31, 32, 50, 55)
9	(21, 37, 39, 43, 43, 67, 68, 69, 72), (9, 18, 24, 37, 39, 45, 48, 67, 77)
13	(11, 27, 30, 32, 37, 56, 59, 62, 64, 64, 64, 65, 85)

Table S3: Adjusted relative risk by day from symptom onset [2] and the derived transmissbility with peak at 5%

Day[1]	Adjusted RR[2]	Transmissbility	Day	Adjusted RR[2]	Transmissbility
-5	0.78	0.0291	1	1.33	0.0496
-4	0.86	0.0321	2	1.27	0.0474
-3	1.02	0.0381	3	1.19	0.0444
-2	1.18	0.0440	4	1.11	0.0414
-1	1.30	0.0485	5	1.05	0.0392
0	1.34	0.0500	5	1.00	0.0373

[1] Day from symptom onset.
[2] RR: relative risk

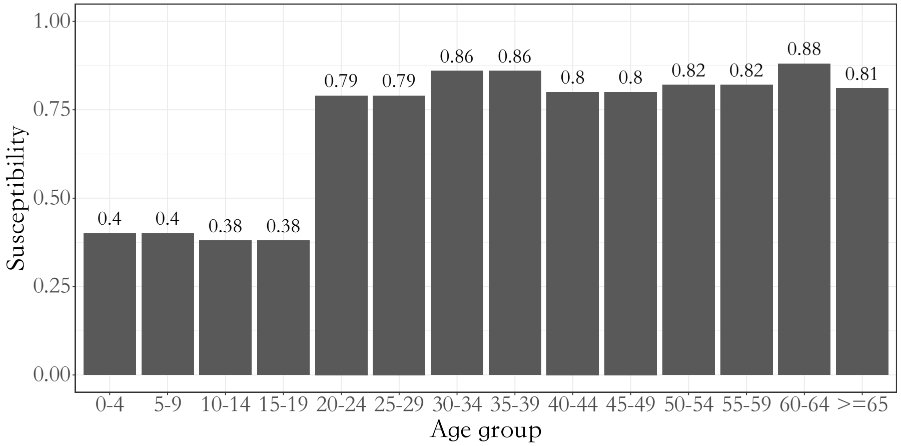

Fig. S1: Susceptibility of individuals of each age group

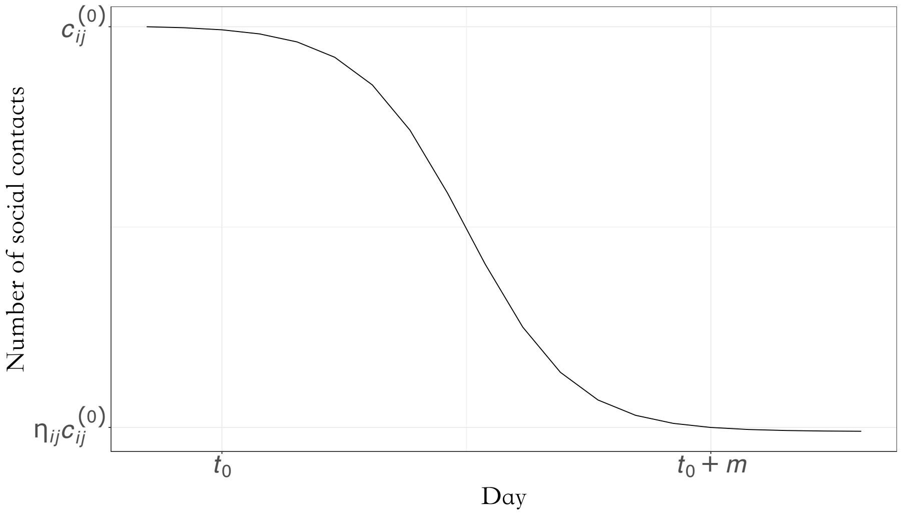

Fig. S2: Contact rates function (S1), where t_0 represents the starting time of the lock-down period, m the duration of the decreasing process, $c_{ij}^{(0)}$ the average number of social contacts between the ith and the jth age group, η_{ij} the percentage of decrease

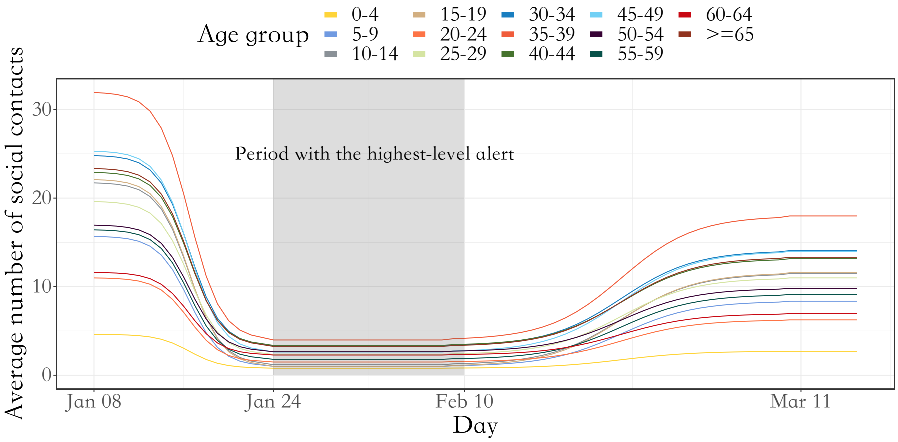

Fig. S3: Average contact number by days that is constructed based on the baseline contact matrix in Shanghai [4] and contact rates function in Fig. S2. Shaded area represents the period between January 24th and February 10th when Zhejiang was adopting highest-level response.

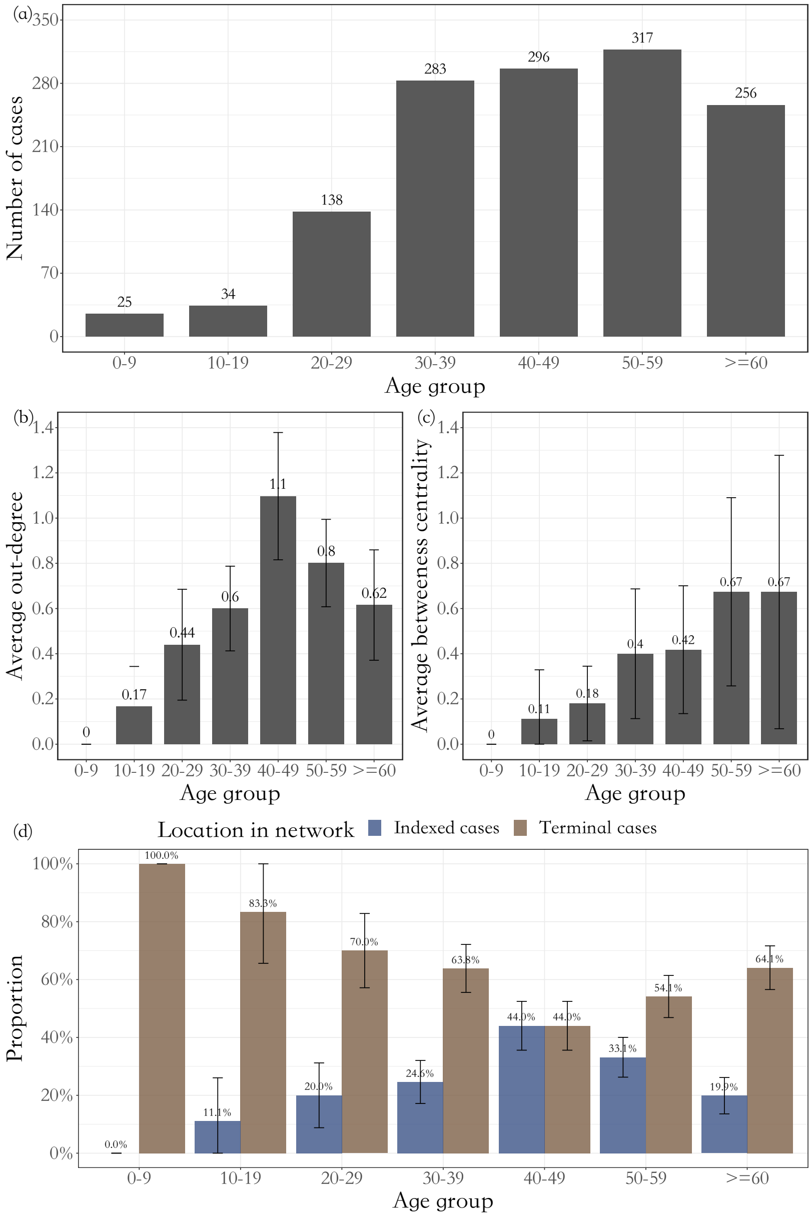

Fig. S4: (a) Number of confirmed cases in different age groups; (b) Average out-degree and 95% confidence interval of cases from each age group; (c) Average betweenness centrality and 95% confidence interval of cases from each age group; (d) Heterogeneity of cases' location in the network related to their age. If a case is the origin of a cluster, it is marked as an indexed case; if a case is positioned as the end of a cluster and thus has no further secondary infection, it is marked as a terminal case (analogous to terminal nodes in a tree).

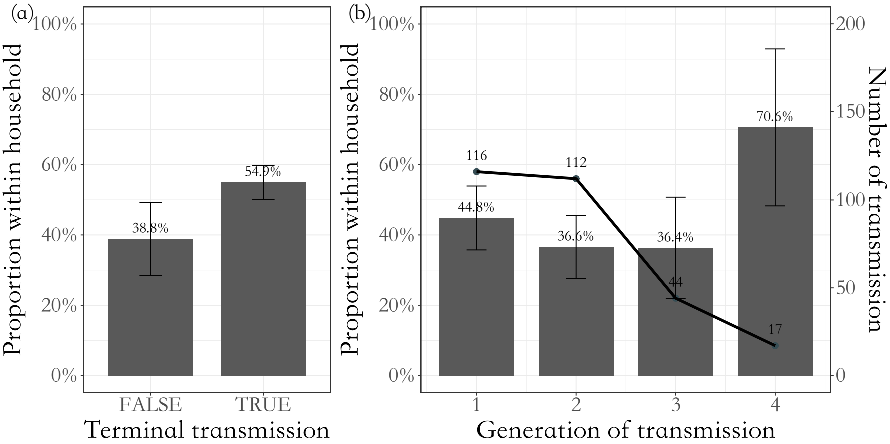

Fig. S5: (a) Proportion of household transmission with respect to whether it resulted in terminal transmission or not; (b) Proportion of household transmission with respect to the generation of transmission. If the infectee is a terminal case, the transmission pointing toward it is marked as a terminal transmission.

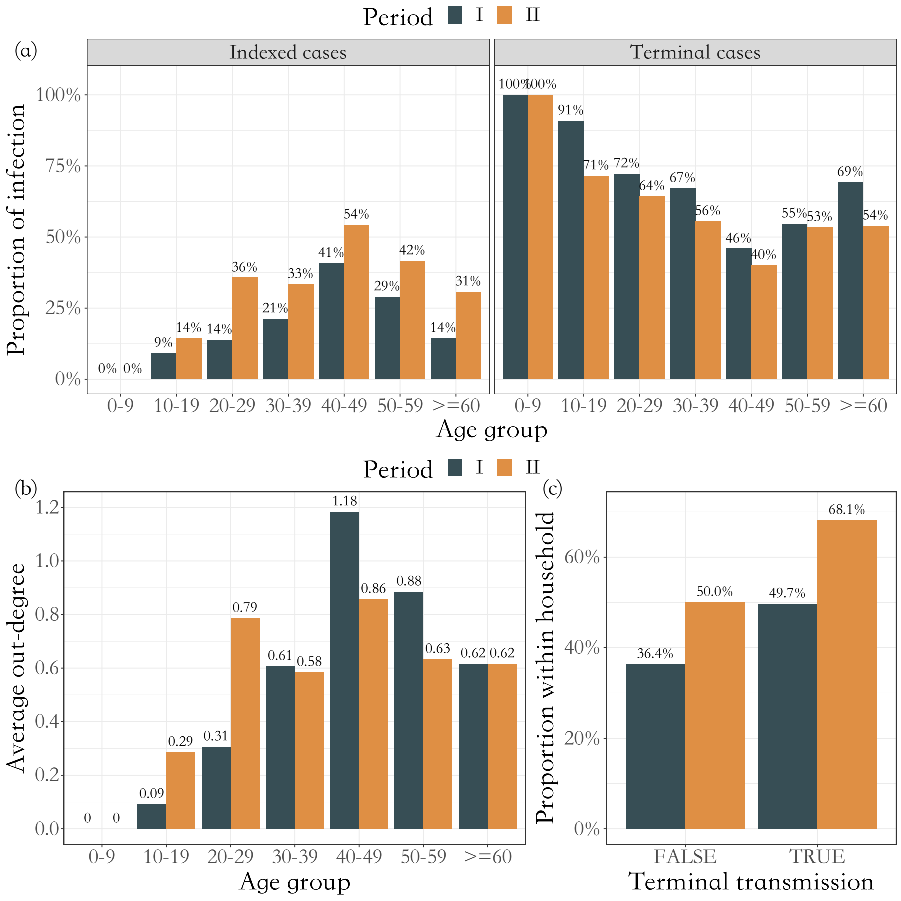

Fig. S6: (a) Heterogeneity of cases' location in the network related to their age by periods; (b) Average out-degree of cases from each age group by periods; (c) Proportion of household transmission with respect to whether terminal transmission or not by periods.

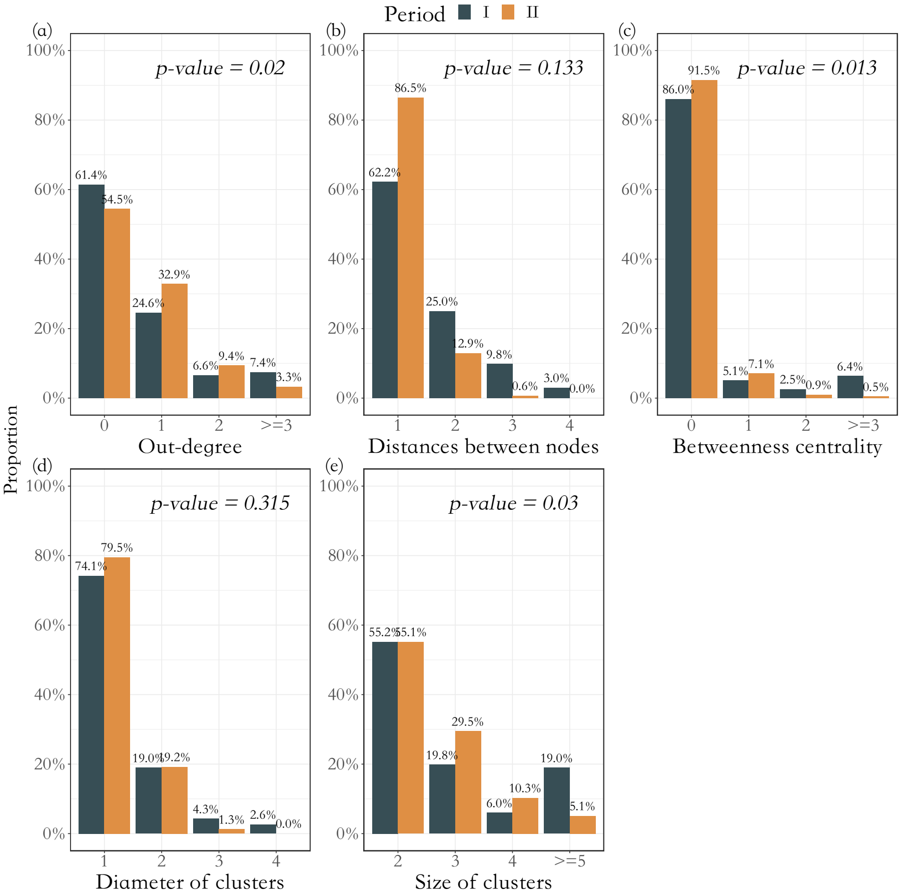

Fig. S7: Distribution of network characteristics across periods. (a) distribution of out-degree of non-singletons; (b) distribution of shortest path length; (c) distribution of betweenness centrality of non-singletons; (d) distribution of diameter of clusters; (e) distribution of size of clusters. For each network attribute, we use Pearson χ^2 test to compare the distribution across periods, and use Benjamini-Hochberg to adjust the p-values.

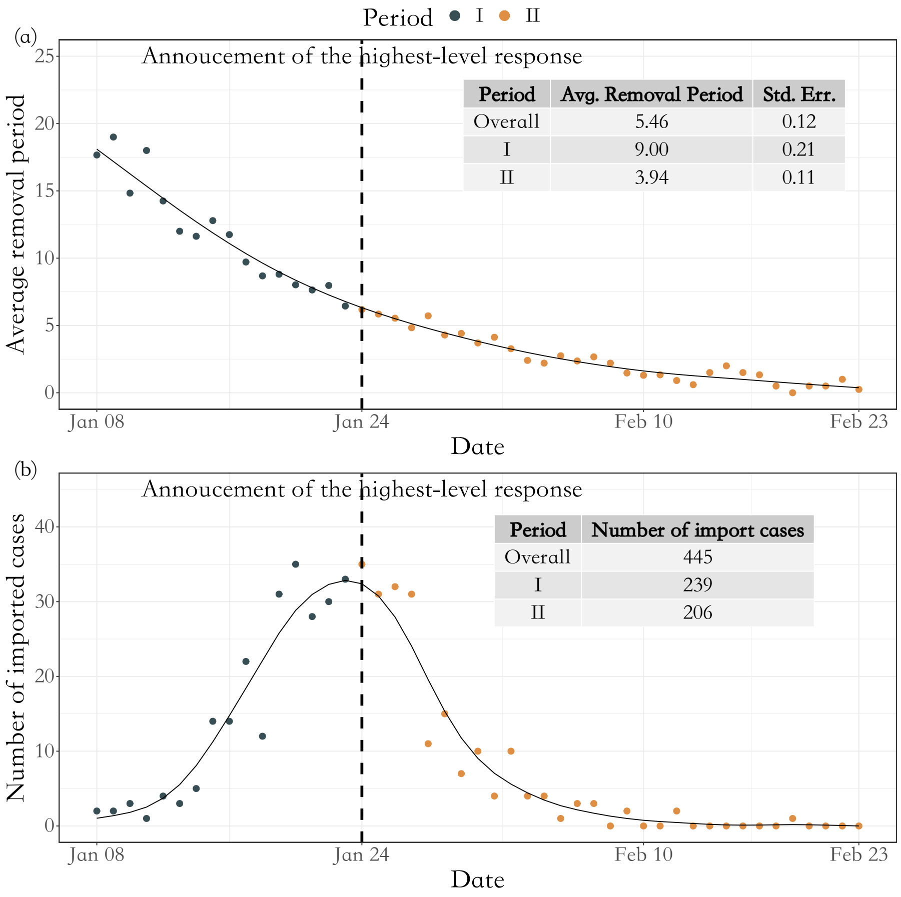

Fig. S8: (a) Average removal periods by date (b) Import cases by date

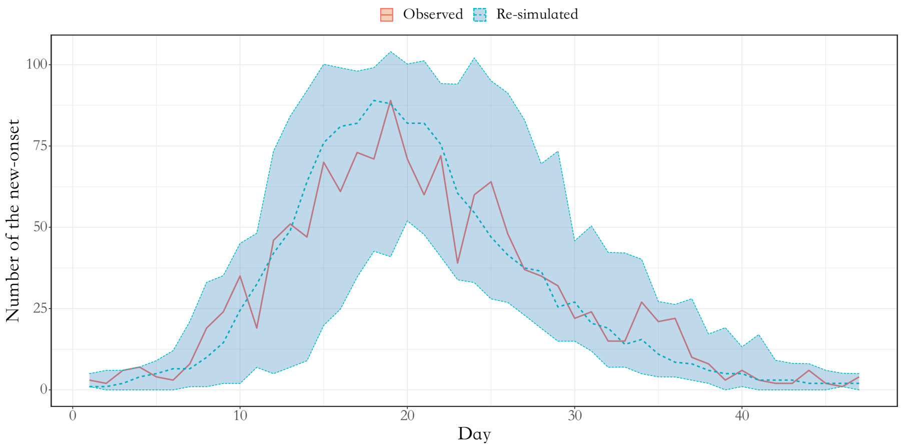

Fig. S9: Daily number of the new-onset for the observed data (line in red) and the real-data-based simulation (line in blue, the median under 200 simulations). The first day is January 8th, 2020. Blue area represents the confidence band under 200 simulations.

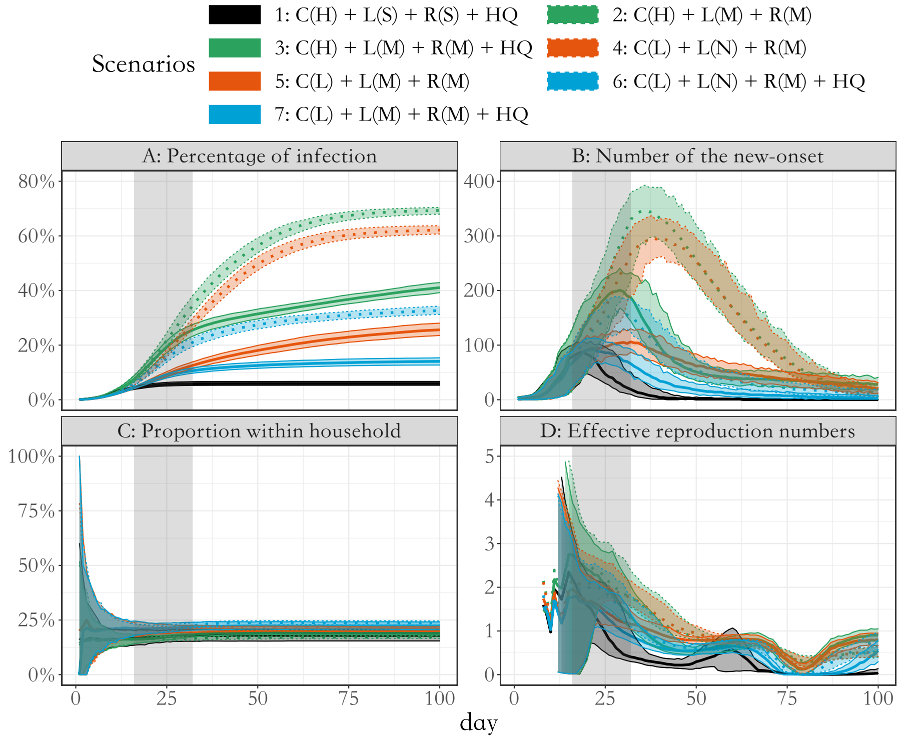

Fig. S10: Confidence band for each scenario in Fig. 7, obtained from 200 simulations.

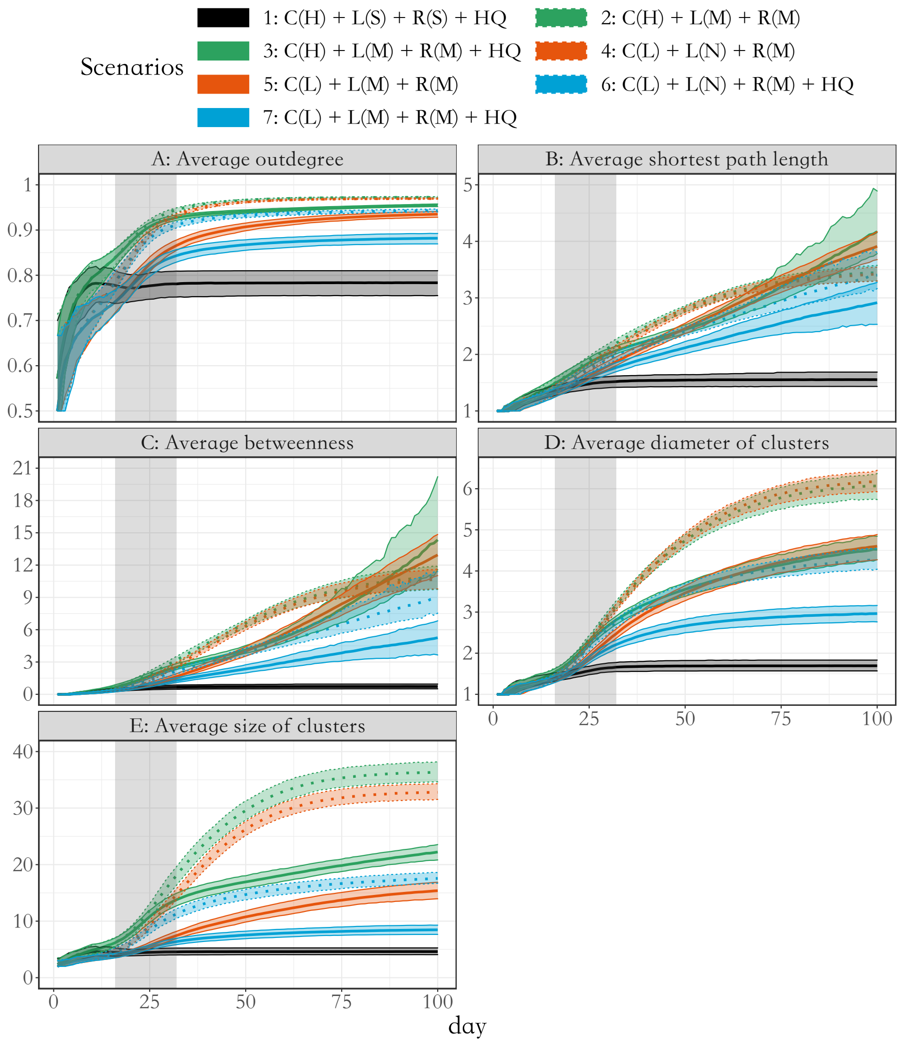

Fig. S11: Confidence band for each scenario in Fig. 8, obtained from 200 simulations.

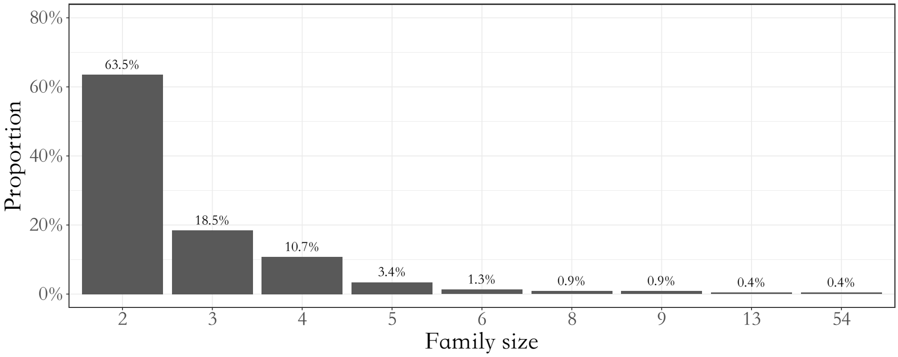

Fig. S12: The distribution of family size. The average family size is 2.94 (SE: 0.24)

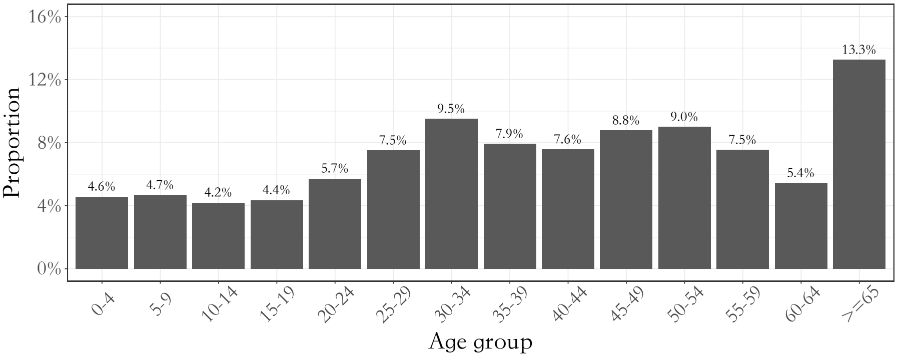

Fig. S13: Age distribution on Zhejiang on the year 2020

References

[1] Shen, Y. *et al.* Community outbreak investigation of sars-cov-2 transmission among bus riders in eastern china. *JAMA Internal Medicine* **180** (12), 1665–1671 (2020) .

[2] Ge, Y. *et al.* Covid-19 transmission dynamics among close contacts of index patients with covid-19: A population-based cohort study in zhejiang province, china. *JAMA Internal Medicine* (2021) .

[3] Mao, G. & Zhang, N. Analysis of average shortest-path length of scale-free network. *Journal of Applied Mathematics* **2013** (2013) .

[4] Zhang, J. *et al.* Changes in contact patterns shape the dynamics of the covid-19 outbreak in china. *Science* **368** (6498), 1481–1486 (2020) .

[5] He, X. *et al.* Temporal dynamics in viral shedding and transmissibility of covid-19. *Nature Medicine* **26** (5), 672–675 (2020) .

[6] Seah, I. Y. J. *et al.* Assessing viral shedding and infectivity of tears in coronavirus disease 2019 (covid-19) patients. *Ophthalmology* **127** (7), 977 (2020) .

[7] Nande, A., Adlam, B., Sheen, J., Levy, M. Z. & Hill, A. L. Dynamics of covid-19 under social distancing measures are driven by transmission network structure. *PLoS Computational Biology* **17** (2), e1008684 (2021) .

[8] Tan, J. *et al.* Transmission roles of symptomatic and asymptomatic covid-19 cases: a modeling study. *medRxiv* 2021.05.11.21257060 (2021) .

[9] Tongjiju, Z. & DiaochaZongdui, G. T. *Zhejiang tongji nianjian-2021 [Statistical yearbook of Zhejiang-2021]* (China Statistic Publishing House,

Zhejiang, China, 2021).

[10] Li, Q. *et al.* Early transmission dynamics in wuhan, china, of novel coronavirus–infected pneumonia. *New England Journal of Medicine* **382** (13), 1199–1207 (2020) .

[11] Nishi, A. *et al.* Network interventions for managing the covid-19 pandemic and sustaining economy. *Proceedings of the National Academy of Sciences* **117** (48), 30285–30294 (2020) .

[12] Davies, N. G. *et al.* Age-dependent effects in the transmission and control of covid-19 epidemics. *Nature Medicine* **26** (8), 1205–1211 (2020) .
